# Aspiration of a dental implant causing airway foreign body in an elderly patient: A case report

**DOI:** 10.1097/MD.0000000000047237

**Published:** 2026-01-16

**Authors:** Fengao Xie, Hancheng Huang, Hanghua Wen, Min Qian, Shuang Han

**Affiliations:** a The First College of Clinical Medical Science, China Three Gorges University, Yichang, Hubei, China; b Yichang Central People’s Hospital, Yichang, Hubei, China.

## Abstract

**Rationale:**

Although the accidental aspiration of dental implant components is exceedingly rare, it is a high-risk complication because it can rapidly cause life-threatening airway obstruction. We present a case to underscore the need for early recognition and prompt airway evaluation when implant parts are lost during dental procedures.

**Patient concerns:**

A 69-year-old woman developed sudden, severe coughing during implant crown placement after an intraoperative crown–abutment assembly became dislodged. The foreign body was not visualized in the oral cavity and was not expelled.

**Diagnosis:**

Non-contrast computed tomography of the chest localized the crown–abutment assembly within the bronchial tree. Imaging confirmed an airway foreign body as the cause of symptoms.

**Interventions:**

Flexible bronchoscopy under local anesthesia was performed. The crown–abutment assembly was grasped and removed using bronchoscopic forceps.

**Outcomes:**

Extraction was successful and uneventful. The patient experienced no postoperative complications.

**Lessons:**

Although the incidence is low, implant-related airway aspiration poses a significant risk and warrants heightened clinical vigilance. When an implant component is lost, clinicians should first consider the high-risk possibility of airway obstruction due to a foreign body and actively rule out airway aspiration before assuming the lower-risk possibility of gastrointestinal entry. This approach improves the timeliness and success of emergency management and enhances patient safety.

## 1. Introduction

With the accelerating global aging process, tooth loss has become an increasingly prominent health issue among the elderly population. Owing to its excellent restorative outcomes and functional recovery, dental implantation has become one of the primary treatment options for tooth replacement in elderly patients.^[1]^Although dental implant technology is well established and is associated with a high overall success rate, the clinical procedures still carry inherent risks that can result in various complications. Among these, accidental aspiration of implant components is an exceptionally rare complication, yet one with potentially catastrophic consequences because it may lead to acute airway obstruction. This case report describes such a rare yet high-risk complication, where a dental implant component became lodged in the airway, and emphasizes that when an implant part is lost, clinicians should first consider and rule out airway aspiration – a high-risk scenario – rather than initially assuming it entered the digestive tract, a relatively low-risk situation, in order to optimize clinical decision-making. This case describes a rare but potentially severe event in which a dental implant component caused airway obstruction.

## 2. Case report

**Figure 1.**
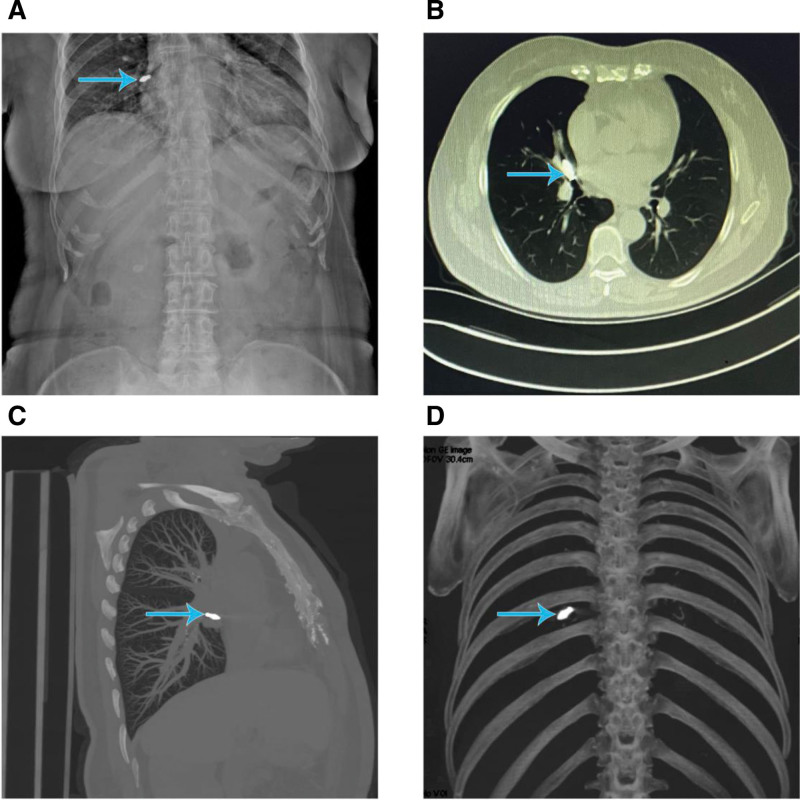
Airway foreign body in a 69-yr-old female patient. The blue arrow indicates the foreign body within the bronchus. (A) Upright abdominal radiograph showing a high-density shadow in the right hypochondriac region. (B–D) Chest CT scan with 3-dimensional reconstruction demonstrating impaction of the crown–abutment assembly at the bifurcation of the right middle lobe bronchus. CT =  computed tomography.

**Figure 2.**
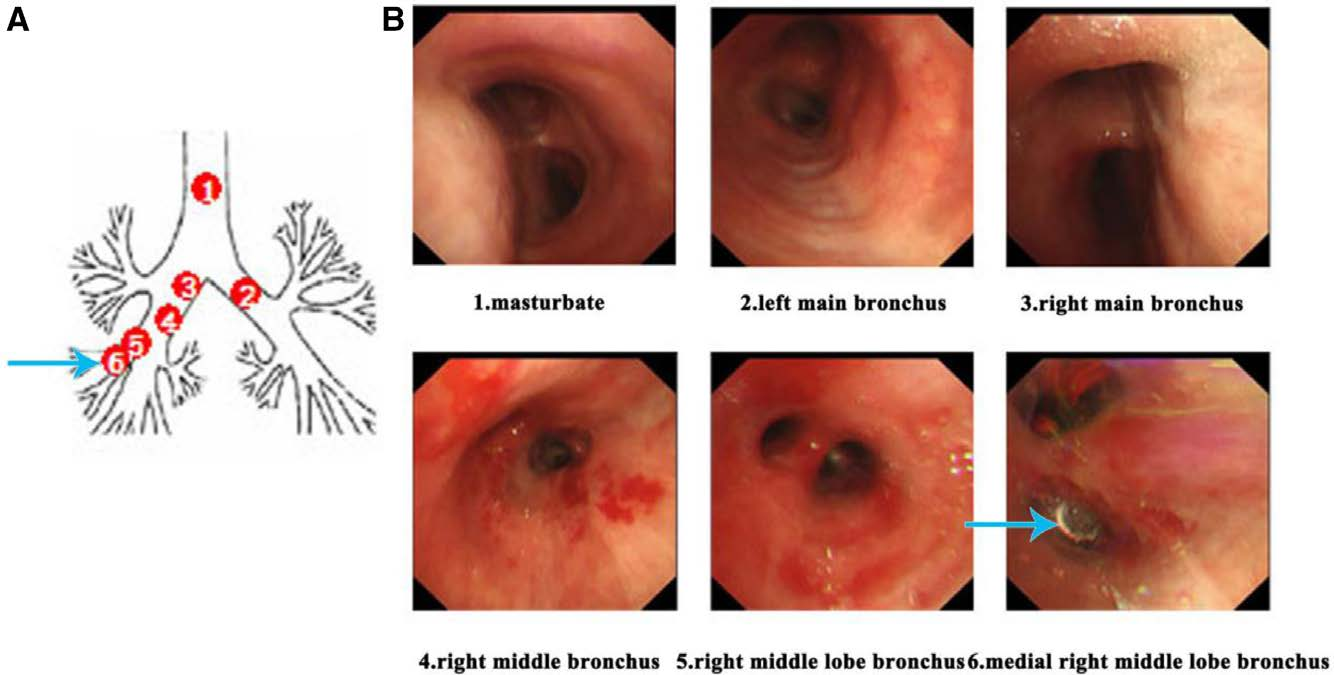
Airway foreign body in a 69-yr-old female patient. The blue arrow indicates the foreign body within the bronchus. (A) Schematic illustration of the bronchus. (B) Endoscopic view of the foreign body under bronchoscopy.

A 69-year-old female patient with no prior cognitive impairment experienced an accidental dislodgement of a crown–abutment assembly during implant crown placement at a dental clinic due to an operator error. The patient immediately developed a severe coughing episode, but the foreign object was neither expelled nor visible in the oral cavity. Despite these choking-like symptoms, the attending dentist, based on clinical experience, presumed that the assembly had been swallowed into the esophagus.After a brief period of observation, the patient’s coughing subsided, and she was discharged without further evaluation or imaging.Within the following 2 days, the patient did not observe passage of the implant component in her stool. To determine its location, she presented to the emergency department of our hospital. An abdominal X-ray revealed a high-density shadow in the right hypochondriac region (Fig. 1A). To further identify the foreign body’s location, a non-contrast chest Computed tomography (CT) scan and 3-dimensional reconstruction were performed (Fig. 1B–D), which revealed that the assembly was lodged at the bifurcation of the right middle lobe bronchus. Bronchoscopy was then carried out under local anesthesia, confirming the presence of the implant assembly impacted in the right middle lobe bronchus (Figs. 2A and F). The foreign body was successfully removed using bronchoscopic forceps. The procedure was uneventful, and no postoperative complications occurred (Figs. 1 and 2).

## 3. Discussion

Airway foreign body refers to the inadvertent entry of external material into the larynx, trachea, or bronchi, resulting in impaired ventilation. With the aging of the population, the incidence of swallowing dysfunction–related foreign body aspiration in the elderly has been increasing annually by approximately 2.3%, becoming an urgent public health concern. As the preferred restorative option for partial edentulism, dental implants have seen widespread adoption – according to the American Dental Association, over 5 million implants are placed annually in the United States, with a utilization rate of 38% among individuals aged 65 years and older. However, the accompanying mechanical complications should not be overlooked.^[2]^Among them, iatrogenic airway foreign bodies represent a severe but preventable complication. Dental procedures account for approximately 12% to 26% of all iatrogenic airway foreign body cases, with the most common objects being dislodged dental crowns (38%), implant instruments (29%), and prosthetic fragments (22%).^[3]^

Due to the anatomical characteristics of the oral cavity, digestive tract, and airway, approximately 0.5% to 2% of ingested objects enter the respiratory tract. Although rare, such cases carry a very high mortality rate. The structural and physical properties of dental implants make them high-risk airway foreign bodies. The dental crown, typically made of ceramic, has a smooth surface, while the titanium abutment possesses a high density (approximately 4.5 g/cm³) and weight, resulting in rapid descent under gravity. The crown–abutment assembly has a dumbbell-shaped configuration, being narrower in the middle and wider at both ends. Owing to the weak adhesion of the airway mucus layer, pressure applied during placement may cause a “piston effect,” propelling the assembly further into the airway.

When the patient is in a supine position, gravity facilitates the rapid sliding of the foreign body along the posterior pharyngeal wall into the airway. The typical implant size (diameter 3–5 mm) closely matches the internal diameter of the right main bronchus. After entering the trachea, the implant tends to enter the right bronchus, which is steeper, shorter, approximately 2 mm wider than the left, and forms a smaller angle (25° vs 45°) with the trachea. Consequently, about 76% of such foreign bodies become lodged at the bronchial bifurcation, leading to complete bronchial obstruction.^[4]^

The clinical manifestations following foreign body aspiration vary widely, ranging from asymptomatic cases to life-threatening conditions. The most common symptoms include coughing, dyspnea, and choking. The longer the foreign body remains in the airway, the greater the extent of airway injury, which may lead to inflammation, granuloma formation, and bronchial stenosis.^[5]^Therefore, timely and accurate diagnosis and management of airway foreign bodies by physicians are of critical importance. Conventional X-ray examination, which is inexpensive and readily accessible, provides rapid imaging reference for both clinicians and patients; however, its sensitivity for detecting nonmetallic foreign bodies is only 40% to 60%.^[6]^ CT represents an excellent diagnostic option. Multidetector spiral CT demonstrates a sensitivity of up to 95% for metallic foreign bodies, and 3-dimensional reconstruction can visualize bronchial impaction, allowing precise assessment of the foreign body’s location as well as its relationship to the airway and surrounding tissues.^[7]^Bronchoscopy often plays a crucial role in the management of impacted airway foreign bodies. Statistics indicate that after foreign body entry into the respiratory tract, 15% to 30% of patients experience temporary symptom relief following a choking episode. This “silent period” can lead clinicians to mistakenly assume that the foreign body has entered the digestive tract, resulting in delayed diagnosis and treatment. The present case exemplifies this clinical pitfall: when a dental implant dislodgement occurs, the sudden onset of severe coughing should not be interpreted as resolution of risk, even if symptoms temporarily subside. Immediate identification of the foreign body’s location is critical for timely diagnosis and management, and imaging should be employed as a mandatory assessment when necessary. This case underscores that airway obstruction is a life-threatening complication that requires heightened clinical attention. We also recommend adopting a stepwise clinical approach, first considering high-risk factors for airway impaction before evaluating lower-risk possibilities, such as entry of the foreign body into the digestive tract.

## Author contributions

**Conceptualization:** Fengao Xie.

**Data curation:** Fengao Xie.

**Resources:** Fengao Xie.

**Writing – original draft:** Fengao Xie, Shuang Han.

**Writing – review & editing:** Fengao Xie, Hancheng Huang, Hanghua Wen, Min Qian, Shuang Han.
